# Effects of a powered ankle-foot prosthesis on kinetic loading of the unaffected leg during level-ground walking

**DOI:** 10.1186/1743-0003-10-49

**Published:** 2013-06-07

**Authors:** Alena M Grabowski, Susan D’Andrea

**Affiliations:** 1 Integrative Physiology Department, University of Colorado Boulder, 354 UCB, Boulder, CO 80309-0354, USA; 2 Department of Veterans Affairs, Eastern Colorado Healthcare System, Denver, CO, USA; 3 Department of Veterans Affairs, Providence VA Medical Center, Providence, RI, USA

**Keywords:** Amputee, Ankle, Biomechanics, Bionic, Gait, Loading rate, Prosthesis, Transtibial, Walking

## Abstract

**Background:**

People with a lower-extremity amputation that use conventional passive-elastic ankle-foot prostheses encounter a series of stress-related challenges during walking such as greater forces on their unaffected leg, and may thus be predisposed to secondary musculoskeletal injuries such as chronic joint disorders. Specifically, people with a unilateral transtibial amputation have an increased susceptibility to knee osteoarthritis, especially in their unaffected leg. Previous studies have hypothesized that the development of this disorder is linked to the abnormally high peak knee external adduction moments encountered during walking. An ankle-foot prosthesis that supplies biomimetic power could potentially mitigate the forces and knee adduction moments applied to the unaffected leg of a person with a transtibial amputation, which could, in turn, reduce the risk of knee osteoarthritis. We hypothesized that compared to using a passive-elastic prosthesis, people with a transtibial amputation using a powered ankle-foot prosthesis would have lower peak resultant ground reaction forces, peak external knee adduction moments, and corresponding loading rates applied to their unaffected leg during walking over a wide range of speeds.

**Methods:**

We analyzed ground reaction forces and knee joint kinetics of the unaffected leg of seven participants with a unilateral transtibial amputation and seven age-, height- and weight-matched non-amputees during level-ground walking at 0.75, 1.00, 1.25, 1.50, and 1.75 m/s. Subjects with an amputation walked while using their own passive-elastic prosthesis and a powered ankle-foot prosthesis capable of providing net positive mechanical work and powered ankle plantar flexion during late stance.

**Results:**

Use of the powered prosthesis significantly decreased unaffected leg peak resultant forces by 2-11% at 0.75-1.50 m/s, and first peak knee external adduction moments by 21 and 12% at 1.50 and 1.75 m/s, respectively. Loading rates were not significantly different between prosthetic feet.

**Conclusions:**

Use of a biomimetic powered ankle-foot prosthesis decreased peak resultant force at slow and moderate speeds and knee external adduction moment at moderate and fast speeds on the unaffected leg of people with a transtibial amputation during level-ground walking. Thus, use of an ankle-foot prosthesis that provides net positive mechanical work could reduce the risk of comorbidities such as knee osteoarthritis.

## Background

There are over one million people in the United States that live with a lower-extremity amputation [1-3] and this number continues to grow appreciably due to the increased prevalence of diabetes. The continued development of carbon-fiber passive-elastic prostheses has enhanced the use of lower-extremity prostheses, but these passive prostheses can only store and return energy. Unlike the biological ankle, passive-elastic prostheses cannot generate non-conservative positive power or work [4-6]. Further, quasi-passive prosthetic ankle joints that employ computer-controlled swing phase position modulation (Proprio Foot Ankle Prosthesis from Össur) propose a measured benefit [7-11], but are incapable of emulating normal biomechanical ankle function during the stance phase of walking. People with a lower-extremity amputation using passive and quasi-passive prostheses continue to experience gait pathologies such as higher metabolic demands, greater kinematic and kinetic leg asymmetries, and reduced self-selected walking speeds [12-17]. Though many potential factors could be causally related to the increased prevalence of musculoskeletal injury in people with a leg amputation, asymmetrical gait patterns such as greater unaffected leg resultant forces and knee moments, have been postulated to increase the risk of unaffected leg musculoskeletal injury, including joint degradation and excessive leg pain [18,19]. Further, when people with a leg amputation use a passive-elastic prosthesis, and walk at faster speeds, they experience greater kinematic and kinetic leg asymmetries, including greater unaffected leg forces [15,20].

People with a transtibial amputation using passive or quasi-passive prostheses display abnormal gait mechanics due in part to the absence of function normally delivered by the muscles surrounding the ankle joint and the absence of ankle range of motion. Most critically, the muscles responsible for plantar flexion of the ankle, the gastrocnemius and soleus, play a key role in human walking [21-23]. These plantar-flexors generate propulsive force during the mid- to late-stance phase and thereby propel the body upward and forward with each walking step [21-23]. Passive-elastic prostheses release less than one-half the mechanical energy, and less than one-eighth the mechanical power normally generated by the soleus and gastrocnemius during the stance phase of level-ground walking at moderate speeds [4-6] and are therefore unable to replicate the function of a biological ankle. Walking at faster speeds requires greater force and power, therefore there are larger kinematic and kinetic discrepancies between passive-elastic prosthetic and biological ankle mechanics [15,20].

The knee external adduction moment (EAM) indicates the load distributed between the medial and lateral compartments of the knee and is strongly associated with the incidence and progression of osteoarthritis in a non-amputee population [24,25]. People with a unilateral transtibial amputation have an increased susceptibility to knee pain and osteoarthritis, especially in their unaffected leg [18,19,26-28], however, they have a decreased prevalence of knee pain in their affected leg [18]. Previous studies have hypothesized that there is a link between the development of osteoarthritis and abnormally high peak knee external adduction moments (EAM) encountered during walking [18,19,24,25,27,29]. Royer and Wasilewski [19] reported significantly higher peak EAM (p = 0.028) in the unaffected leg of subjects with a unilateral transtibial amputation (0.55 ± 0.18 Nm/kg) compared to their affected leg (0.38 ± 0.22 Nm/kg). Similar findings have been reported by Lloyd and colleagues [30]. When faced with knee pain in their unaffected leg, people with an amputation may reduce or forgo recreation, social, and family activities compared to non-amputees [18].

One of the major factors contributing to the prevalence of knee osteoarthritis in the unaffected leg of people with a unilateral transtibial amputation is believed to be related to the asymmetrical loading of the joint. Greater forces and loading rates observed in the unaffected leg may add to the risk of knee osteoarthritis. Kinetic loading rates have been used in previous studies to distinguish people with musculoskeletal injury from those without injury. Prior research has shown that people with a history of musculoskeletal running injuries such as plantar fasciitis and tibial stress fractures have greater vertical ground reaction force loading rates, defined as the slope of the vertical ground reaction force curve from 20–80% of heel-strike to first peak vertical force, compared to uninjured runners [31,32]. Mundermann et al. [29] found that vertical ground reaction force loading rates in patients with knee osteoarthritis during walking were elevated by 50.1% compared with those of matched uninjured subjects. However, to our knowledge, no one has compared resultant ground reaction force loading rates in subjects with an amputation. Further, we know of no studies that have calculated the loading rate of the knee EAM. Presumably, reductions in peak knee EAM would correlate with reductions in EAM loading rate.

During a single level-ground walking stride, both legs must perform positive and negative work on the center of mass (COM) to transition between steps [33-35]. Individual leg work equals the time integral of the dot product of the leg’s ground reaction force and the COM velocity vector during the step-to-step transition, or double support phase, of walking. Step-to-step transitions are optimal when the positive push-off work and negative collision work are equal in magnitude [36,37]. When people with a unilateral amputation use a passive-elastic prosthesis, their affected trailing leg performs insufficient positive work during step-to-step transitions [4-6] and thus their unaffected leading leg compensates by absorbing a greater amount of negative work [38,39]. Further, the work absorbed by the unaffected leading leg increases with faster walking speeds [38]. Previous analytical studies of walking suggest that the application of a push-off force by the trailing leg just prior to the leading leg heel-strike is the most efficient method of decreasing the large negative work absorbed during step-to-step transitions [35,37]. Motivated in part by this biomechanical model finding, a novel powered ankle-foot prosthesis, the BiOM, now commercially-available from iWalk, Inc., has been designed to generate biomimetic ankle power [40-46] and allows people with transtibial amputations to achieve normative preferred walking speeds, metabolic demands, and step-to-step transition work across a wide range of speeds compared to non-amputees [38]. This powered prosthesis provides net positive work during step-to-step transitions, thereby increasing trailing leg work, and decreasing leading leg collision work compared to a passive-elastic prosthesis [38]. Use of a prosthesis that generates normative ankle power could decrease kinetic asymmetries between the affected and unaffected legs of people with a unilateral transtibial amputation. By providing adequate push-off work via a powered ankle-foot prosthesis, collision work on the unaffected leg is reduced, which presumably reduces the peak resultant force and first peak knee EAM.

We seek to determine the kinetic effects of a powered ankle-foot prosthesis on the unaffected leg of people with a unilateral transtibial amputation over level-ground across the full range of walking speeds. We hypothesize that, compared to using a passive-elastic prosthesis, people with a unilateral transtibial amputation using a powered ankle-foot prosthesis will have lower peak resultant ground reaction forces, peak external knee adduction moments, and the associated loading rates applied to their unaffected leg during level-ground walking over a range of speeds. We also hypothesize that compared to non-amputees, people with a unilateral transtibial amputation using a powered ankle-foot prosthesis will have equivalent peak resultant ground reaction forces, peak external knee adduction moments, and the associated loading rates applied to their unaffected leg during level-ground walking over a range of speeds.

## Methods

### Study participants

Seven people with a unilateral transtibial amputation and seven age-, sex-, height- and weight-matched non-amputees gave informed written consent according to the Department of Veterans Affairs Research Service Providence VA Medical Center Institutional Review Board (IRB # 00001402) prior to participation. All research was conducted in compliance with the Helsinki Declaration. Subjects with an amputation were at least two years post-amputation, had an amputation due to trauma, and were at or above a K3 Medicare Functional Classification Level. All subjects had no known cardiovascular, pulmonary, or neurological disease or disorder, and no additional musculoskeletal injury (Table 1). Prior to participation, subjects with an amputation were evaluated by a certified prosthetist that quantified and confirmed their level of amputation and disability. All subjects with an amputation used conventional passive-elastic prostheses to walk during their normal daily activities.

**Table 1:** Anthropometric characteristics

**Amputee**	**Age**	**Height**	**Mass**	**Leg length**	**Years since amputation**	**Prosthesis**
	**(yrs)**	**(m)**	**(kg)**	**(m)**		
1	37	1.89	90.0	1.02	17	Ossur Flex-Foot
						VSP
2	45	1.74	92.7	0.93	19	College Park
						Venture
3	50	1.74	90.7	0.92	39	Freedom Innov. Renegade
4	50	1.80	106.7	0.98	31	Ossur Flex-Foot
						Re-Flex VSP
5	39	1.94	111.0	1.02	20	Ossur Flex-Foot
						Vari-Flex EVO
6	42	1.82	112.7	1.00	20	Otto Bock
						Axtion
7	51	1.73	92.6	0.95	2	Ohio Willow Wood Limb Logic
**Amputee**	**45 (6)**	**1.81 (0.08)**	**99.5 (10.2)**	**0.97 (0.04)**	**21.1 (11.6)**	
**Avg. (S.D.)**						
**Control**	**48 (7)**	**1.86 (0.06)**	**97.7 (11.9)**	**1.02 (0.03)**		
**Avg. (S.D.)**						

All subjects with an amputation were at a K3 level of ambulation, had an amputation due to trauma, and were male. Non-amputee subjects (Control) were age-, sex-, height-, and weight-matched.

### Powered ankle-foot prosthesis

**Figure 1:**
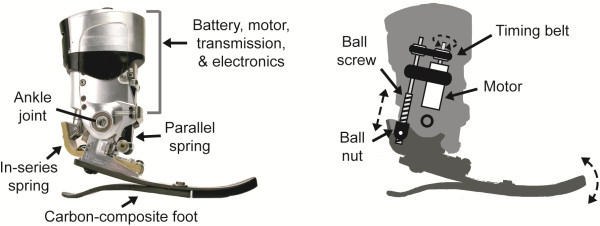
**Powered ankle-foot prosthesis.** The powered prosthesis uses a series-elastic actuator comprised of a brushless 200 Watt DC motor, ball screw transmission, and carbon-composite series leaf spring. The actuator is capable of performing non-conservative positive work about the ankle joint. The motor, transmission, and electronics are contained above the prosthetic ankle joint, and a modular Lithium-Polymer battery is housed most proximal to the ankle joint. The base of the prosthesis consists of a carbon-composite leaf spring, which adds compliance at the heel and forefoot.

The powered ankle-foot prosthesis (Figure 1) employs both passive and motorized elements to more closely emulate human ankle-foot functions. Like the biological ankle, the device generates net positive work during the stance phase and biological levels of mechanical power during terminal stance [42]. The prosthesis uses a series-elastic actuator, configured with a brushless motor and ball screw transmission in series with a carbon composite leaf spring, to store and release motor energy; thus improving efficiency and power output (Figure 1). Like state-of-the-art passive and quasi-passive ankles, the powered prosthesis features a carbon-composite foot at its base for added compliance. All electronics are encapsulated within a single housing. A modular Lithium-Polymer battery powers the motor and slides into an external compartment (Figure 1). The mass of the prosthesis is approximately 2.0 kg including the battery, similar to the mass of a biological foot and partial shank of an 80 kg male [47].

Feedback data from prosthetic ankle torque sensors ensure that the powered prosthesis achieves biomimetic function by constantly varying actuator torque and impedance throughout the gait cycle to match biological norms. Biologically-inspired control schemes govern the behavior of the device, enabling proper timing and magnitude of ankle power for a wide range of walking speeds [45,46]. The adaptive ankle controller employs positive torque feedback reflex control, using sensory information from both the actuator torque and the net torque on the ankle joint.

### Procedure

Subjects with an amputation completed two randomized experimental walking sessions; one using their own passive-elastic prosthesis and one using the powered ankle-foot prosthesis. Non-amputee subjects completed one experimental session. All data were collected at the Gait and Motion Analysis Laboratory of the Providence, RI VA Medical Center, Center for Restorative and Regenerative Medicine. Before experimental sessions with the powered ankle-foot prosthesis, subjects with an amputation completed a fitting and acclimation session of at least 2 hours. During this session, a certified prosthetist ensured that the prosthesis was properly fit and aligned. Then, each subject walked at 0.75, 1.00, 1.25, 1.50, and 1.75 m/s, while we adjusted the stiffness and power delivery of the powered prosthesis so that prosthetic ankle angle at toe-off and net positive mechanical work, the time integral of ankle power during the entire stance phase, matched average biological ankle data [23,48] within two standard deviations of the mean [38] (Table 2). The prosthesis was not tuned to a specific walking speed, but rather the same set of control parameters were used across all speeds.

**Table 2:** Dynamic behavior of the powered prosthesis

**Speed**	**Toe-off angle (deg)**		**Ankle net work (J/kg)**		**Peak ankle power (W/kg)**	
**(m/s)**	**Control**	**Powered**	**Control**	**Powered**	**Control**	**Powered**
0.75	12.0 ± 4.6	13.2 ± 2.5	-0.03 ± 0.08	0.12 ± 0.06*	1.4 ± 0.5	1.3 ± 0.3
1.00	15.3 ± 4.7	15.3 ± 2.3	0.02 ± 0.07	0.14 ± 0.07*	2.2 ± 0.6	1.7 ± 0.4
1.25	16.8 ± 4.4	16.7 ± 1.9	0.07 ± 0.06	0.17 ± 0.09*	2.8 ± 0.6	2.6 ± 0.4
1.50	18.2 ± 5.9	18.6 ± 1.6	0.12 ± 0.09	0.22 ± 0.07*	3.4 ± 0.6	3.8 ± 0.5
1.75	19.1 ± 3.5	19.0 ± 1.2	0.16 ± 0.06	0.25 ± 0.08	4.2 ± 0.7	4.2 ± 0.6

Average ± S.D. ankle angle at toe-off, net mechanical work during the entire stance phase, and peak mechanical power for subjects with an amputation using a powered ankle-foot prosthesis (Powered) compared to non-amputees (Control) across walking speeds. We used data from sensors within the prosthetic ankle to compute toe-off angle and net work from the powered prosthesis. We used inverse dynamics to compute data from non-amputees and peak power for both groups. * indicates a significant difference (P ≤ 0.05) between subjects with an amputation using the powered prosthesis and non-amputees.

Prior to each data collection session, we placed reflective markers on the following lower body anatomical landmarks of each leg: anterior superior iliac spine, posterior superior iliac spine, iliac crest, greater trochanter, medial and lateral femoral condyles, medial and lateral malleoli, 1st and 5th metatarsal heads, base of the 5th metatarsal, calcaneus, clusters of at least 3 markers along the thigh and shank segments, and over the 7^th^ cervical vertebrae of each subject. Marker placements for the affected leg were matched to those of the unaffected leg. During each experimental session, subjects walked 0.75, 1.00, 1.25, 1.50, and 1.75 m/s across a 10 m instrumented level walkway. We used a 3-D motion analysis system (Qualysis Oqus, Gothenburg, Sweden) and two force platforms (Advanced Medical Technology Incorporated, Watertown, MA) embedded in the walkway to simultaneously measure ground reaction forces at 1000 Hz and kinematics at 100 Hz during each set of experimental trials. We analyzed 3 trials from each subject at each velocity and only considered walking trials where the participant’s velocity, measured as the horizontal distance per unit time of the marker placed over the 7^th^ cervical vertebrae, was within 0.10 m/s of the target velocity, and where each foot made full contact with each force plate. We asked subjects to repeat the walking trials until they met these criteria.

We digitized the reflective marker positions using motion tracking software (Qualysis Track Manager, Gothenburg, Sweden). Then we filtered the marker data with a 6 Hz Butterworth low-pass filter and used inverse dynamics (Visual 3D, C-Motion, Inc.) to determine sagittal plane ankle joint power over the entire stance phase for the powered prosthetic ankle and the biological ankle, and frontal plane knee moments over the entire stance phase for the unaffected legs of all subjects. We calculated biological ankle power and powered prosthetic ankle power using inverse dynamics. Because the powered prosthesis has a mass that is equivalent to the mass of the biological foot and partial shank of an 80 kg male [42], and the center of ankle rotation is similar to that of a biological ankle, we assumed that an inverse dynamics approach was appropriate to calculate powered prosthetic ankle power. We created a custom Matlab program (Matlab, Mathworks, Natick, MA) to calculate resultant force, or the magnitude of the ground reaction force vector, and to detect stance phases using a 10 N resultant force threshold. Then we up-sampled the joint kinematic and kinetic data, combined them with the ground reaction force data and normalized all the data to a step.

We calculated the impact peak of the resultant ground reaction force as the maximum force during the first half of the stance phase and calculated the first peak knee external adduction moment (EAM) as the maximum EAM during the first half of the stance phase. We calculated average loading rates of the resultant ground reaction forces and the EAMs from 20 to 80% of the time between foot-strike and the first peak of each variable [31]. This portion of each curve indicates the linear loading response of the resultant force and the external adduction moment. We calculated the average loading rate from the change in force or EAM divided by change in time during this period.

### Statistics

We compared unaffected leg peak resultant ground reaction force, peak knee EAM, and the corresponding loading rates from subjects with a unilateral transtibial amputation using a passive-elastic prosthesis to the same subjects using a powered prosthesis with repeated-measures ANOVAs. We also compared these data from subjects with a unilateral transtibial amputation using a passive or powered prosthesis to non-amputees with one-way ANOVAs. And we compared ankle toe-off angle, net positive work, and peak power from subjects with a unilateral transtibial amputation using a powered prosthesis to non-amputees with one-way ANOVAs. Significant differences were further analyzed with a Tukey HSD follow-up procedure and detected as P ≤ 0.05. We performed post-hoc statistical power analyses on our data with n = 7 for peak resultant force, peak EAM, and the respective loading rates for subjects using the powered prosthesis [49]. We averaged the statistical powers across all the velocities and calculated an average statistical power of 0.96 to detect a 15 per cent difference and 0.84 to detect a 10 per cent difference in peak resultant force, 0.57 to detect a 15 per cent difference and 0.35 to detect a 10 per cent difference in peak EAM, 0.39 to detect a 15 per cent difference and 0.23 to detect a 10 per cent difference in resultant force loading rate, and 0.37 to detect a 15 per cent difference and 0.22 to detect a 10 per cent difference in EAM loading rate. Thus, we believe we had strong statistical power for detecting differences in peak resultant forces, moderate statistical power for detecting differences in peak EAMs, and insufficient statistical power for detecting differences in loading rates.

## Results

Use of a powered ankle-foot prosthesis reduced the peak resultant forces on the unaffected leg of subjects with an amputation at slow and moderate walking speeds compared to use of a passive-elastic prosthesis. The impact peaks of the resultant ground reaction forces (GRFs) from the unaffected leg were significantly lower when subjects with an amputation used the powered prosthesis compared to using their own passive-elastic prosthesis at speeds of 0.75, 1.00, 1.25, and 1.50 m/s (P = 0.04, 0.01, 0.05, and 0.04, respectively; Table 3). On average, across speeds of 0.75-1.50 m/s, the impact peak resultant GRFs on the unaffected leg were 6.6% lower for subjects using the powered prosthesis compared to using their passive-elastic prosthesis. The average resultant GRF loading rates of the unaffected leg were between 4-13% lower when subjects with an amputation used the powered prosthesis compared to their passive-elastic prosthesis across walking speeds of 0.75-1.75 m/s, but these loading rates were not significantly different (Table 3).

**Table 3:** Unaffected leg resultant ground reaction force impact peaks and loading rates

**Speed**	**Unaffected leg 1**^**st **^**peak GRF (N/kg)**				**Unaffected leg GRF rate (N/kg/s)**			
**(m/s)**	**Passive**	**Powered**	**% Diff**	**Control**	**Passive**	**Powered**	**% Diff**	**Control**
0.75	9.97 ± 0.21*^	9.76 ± 0.13	-2.1	9.79 ± 0.27	71.7 ± 36.6	68.8 ± 26.2	-4.0	49.2 ± 16.5
1.00	10.39 ± 0.40*	9.75 ± 0.22	-6.2	9.86 ± 0.37	87.0 ± 39.2	82.5 ± 23.1	-5.2	73.5 ± 15.0
1.25	11.33 ± 0.67*^	10.52 ± 0.75	-7.2	10.62 ± 0.39	118.7 ± 41.9^	103.7 ± 28.8^	-12.6	79.6 ± 7.4
1.50	12.77 ± 1.10*^	11.41 ± 1.28	-10.7	11.58 ± 0.75	137.1 ± 53.2	123.6 ± 22.9	-9.8	104.5 ± 18.9
1.75	13.87 ± 1.24^	13.42 ± 1.70	-3.3	12.32 ± 0.41	176.6 ± 46.8	160.5 ± 44.6	-9.1	151.6 ± 43.5

Average ± S.D. resultant ground reaction force impact peaks and resultant ground reaction force loading rates of each subject with an amputation using a passive-elastic (Passive) or powered (Powered) prosthesis, and non-amputee subjects (Control) across a range of walking speeds. The decreases in peak GRFs and loading rates between the passive-elastic and powered prostheses are shown as a percentage difference (% Diff). * indicates a significant difference (P ≤ 0.05) between subjects with an amputation using the passive-elastic versus powered prostheses. ^ indicates a significant difference (P ≤ 0.05) between subjects with an amputation and non-amputees (Control). P-values for GRF loading rates between subjects with an amputation using the passive-elastic versus powered prostheses were 0.81, 0.70, 0.27, 0.36, and 0.14 at speeds of 0.75, 1.00, 1.25, 1.50, and 1.75 m/s, respectively.

Use of a powered ankle-foot prosthesis reduced the external adduction moment (EAM) on the unaffected knee of subjects with an amputation compared to use of a passive-elastic prosthesis at the two fastest walking speeds. The unaffected leg first peak knee EAM was significantly lower when subjects with an amputation used the powered prosthesis compared to their passive-elastic prosthesis during walking at 1.50 and 1.75 m/s (P = 0.03 and 0.05, respectively; Table 4). Peak EAM was 20.6% and 12.2% lower for subjects using a powered compared to a passive-elastic prosthesis at 1.50 m/s and 1.75 m/s, respectively. We did not find statistical differences in peak EAM at speeds of 0.75, 1.00, and 1.25 m/s (P = 0.36, 0.88, and 0.44, respectively). The average unaffected knee EAM loading rates were 5-22% lower when subjects with an amputation used the powered prosthesis compared their passive-elastic prosthesis across walking speeds of 0.75-1.75 m/s, but these loading rates were not significantly different (Table 4).

**Table 4:** Unaffected leg peak knee EAMs and loading rates

**Speed**	**Unaffected leg 1**^**st **^**peak EAM (Nm/kg)**				**Unaffected leg EAM rate (Nm/kg/s)**			
**(m/s)**	**Passive**	**Powered**	**% Diff**	**Control**	**Passive**	**Powered**	**% Diff**	**Control**
0.75	0.41 ± 0.13	0.39 ± 0.08	-5.1	0.39 ± 0.13	1.95 ± 0.85	1.84 ± 0.42	-5.4	1.74 ± 0.88
1.00	0.42 ± 0.12	0.42 ± 0.09	-0.8	0.34 ± 0.14	2.73 ± 1.10	2.24 ± 0.68	-17.9	1.86 ± 1.08
1.25	0.50 ± 0.14	0.47 ± 0.10	-5.5	0.38 ± 0.11	3.89 ± 1.43	3.38 ± 1.02	-12.9	2.64 ± 1.15
1.50	0.61 ± 0.16*	0.49 ± 0.06	-20.6	0.44 ± 0.14	4.79 ± 1.55	3.73 ± 0.82	-22.1	3.72 ± 1.79
1.75	0.68 ± 0.16*	0.60 ± 0.14	-12.2	0.50 ± 0.15	6.01 ± 1.60	5.11 ± 1.66	-15.0	4.49 ± 1.29

Average ± S.D. first peak knee EAMs and loading rates of the unaffected leg of each subject with an amputation using a passive-elastic (Passive) or powered (Powered) prosthesis, and non-amputee subjects (Control) across a range of walking speeds. * indicates a significant difference (P ≤ 0.05) between subjects with an amputation using the passive-elastic versus powered prostheses. P-values for EAM loading rates between subjects with an amputation using the passive-elastic versus powered prostheses were 0.60, 0.07, 0.14, 0.07, and 0.17, at speeds of 0.75, 1.00, 1.25, 1.50, and 1.75 m/s, respectively.

**Figure 2:**
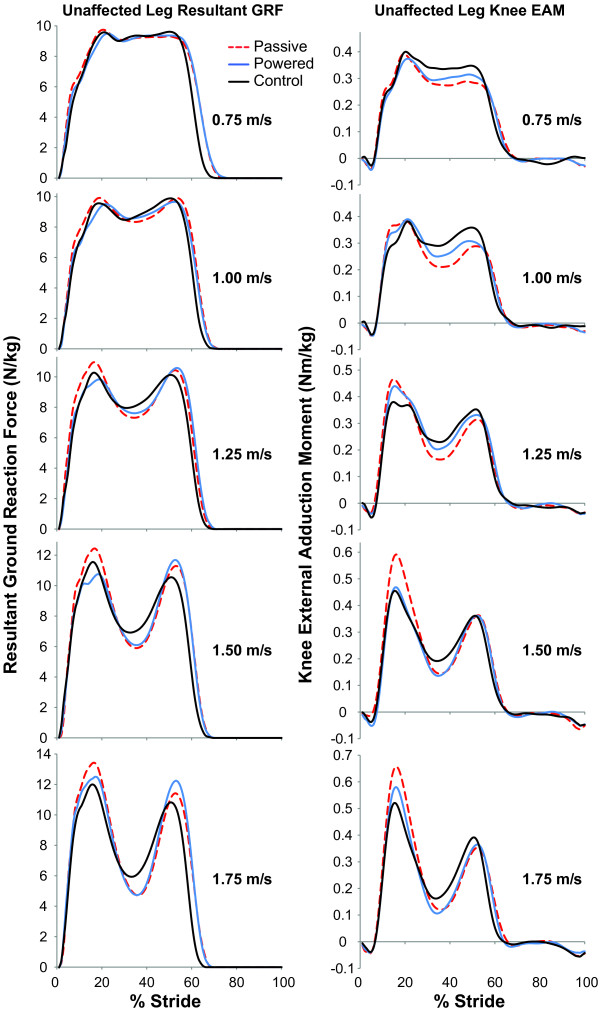
**Average unaffected leg resultant ground reaction force (GRF) and knee external adduction moment (EAM).** Dashed red lines indicate GRFs (left column) and EAMs (right column) of the unaffected leg while subjects walked using a passive-elastic prosthesis (Passive) across a range of speeds. Blue lines represent GRFs (left column) and EAMs (right column) of the unaffected leg while subjects walked using the powered prosthesis (Powered). Black lines represent GRFs (left column) and EAMs (right column) of non-amputees (Control). The average of three steps from all subjects is shown. Data are plotted versus percentage of a stride, where 0% occurs at heel strike.

We found that when subjects with an amputation used the powered prosthesis compared to their passive-elastic prosthesis, they reduced the peak resultant forces and EAMs on their unaffected leg across a range of walking speeds, but their GRF and EAM traces did not directly match those of non-amputees (Figure 2). Compared to non-amputees, subjects with an amputation that used their own passive-elastic prosthesis had greater peak resultant forces on their unaffected leg at walking speeds of 0.75, 1.25, 1.50, and 1.75 m/s (P = 0.04, 0.04, 0.05, and 0.03, respectively; Table 3). However, when subjects with an amputation used the powered prosthesis, the peak resultant forces on their unaffected leg were not significantly different from non-amputees. At one speed, 1.25 m/s, the resultant GRF loading rate for non-amputees was significantly lower than both prosthetic conditions in subjects with an amputation (P = 0.03 and 0.04 for passive-elastic and powered prostheses, respectively). Even though peak resultant forces were different between subjects with an amputation using a passive-elastic prosthesis and non-amputees, there were no significant differences in peak knee EAM or EAM loading rates between these groups (Table 4).

## Discussion

Our results partially confirm our hypotheses. Compared to using a passive-elastic prosthesis, people with a unilateral transtibial amputation using a powered ankle-foot prosthesis had significantly lower peak resultant GRFs at 0.75-1.50 m/s, and peak knee EAMs at 1.50 and 1.75 m/s applied to their unaffected leg during level-ground walking. Though there were no statistical differences in unaffected leg loading rates for GRFs and knee EAMs, there were trends of reduced loading rates when subjects used the powered prosthesis compared to the passive-elastic prosthesis. The lack of statistical differences in loading rates may be due to the high variability in our loading rate data (Tables 3 and 4). A greater number of subjects and more than three steps per condition (e.g. using an instrumented treadmill) would increase the statistical power and likely reduce the variability of the loading rates, thus confirming or refuting expected differences in unaffected leg loading rates between prostheses.

There is a greater prevalence of knee osteoarthritis in the unaffected compared to the affected leg of people with an amputation using a passive-elastic prosthesis [18,19,26-28]. We found that the unaffected leg peak knee EAMs were greater when subjects used a passive-elastic compared to a powered prosthesis, which is likely due to the limited push off provided by a passive prosthesis. Morgenroth et al. [39] has suggested that the first peak knee EAM scales with net positive ankle work and found that a passive-elastic prosthesis with the greatest net positive ankle push-off work resulted in the lowest unaffected leg first peak knee EAM compared to prostheses with little ankle push-off work.

 At speeds of 0.75 and 1.00 m/s, the unaffected leg first peak knee EAMs were similar between subjects with an amputation using a passive-elastic prosthesis and a powered prosthesis, and non-amputees. At these slow walking speeds, the passive-elastic and powered prostheses, as well as the biological ankle behave in a spring-like manner, where the net mechanical work is nearly zero across the entire stance phase (Table 2). Whereas, at the two fastest speeds of 1.50 and 1.75 m/s, there were significant differences in unaffected leg peak knee EAMs (Table 4, Figure 2). The significantly greater unaffected leg peak knee EAM in subjects with an amputation using a passive-elastic prosthesis is likely due to the limited amount of push-off work provided by the passive prosthesis [38]. Thus, the reason for differences in unaffected leg peak knee EAM is likely due to the net positive work performed at faster speeds by the powered prosthesis (Table 2).

Researchers have hypothesized that peak resultant force and knee EAM are factors that may be linked to common medical complications such as knee osteoarthritis [27], thus we believe that a powered ankle-foot prosthesis may reduce the risk of these complications by decreasing unaffected leg peak resultant forces and knee EAM over a range of walking speeds. Previous studies and models have shown the importance of powered plantar flexion during the walking gait cycle [39]. People with unilateral transtibial amputations using passive-elastic prostheses employ compensatory mechanisms such as an increased dependence on the unaffected leg during walking that result in greater peak forces on the unaffected leg compared to the affected leg [15,20,50]. Our results show that use of a powered ankle-foot prosthesis decreases the unaffected leg peak impact resultant force and loading rate. This suggests that increased powered plantar flexion may mitigate some of the compensatory mechanics used by people with unilateral transtibial amputation over a wide range of walking speeds.

**Figure 3:**
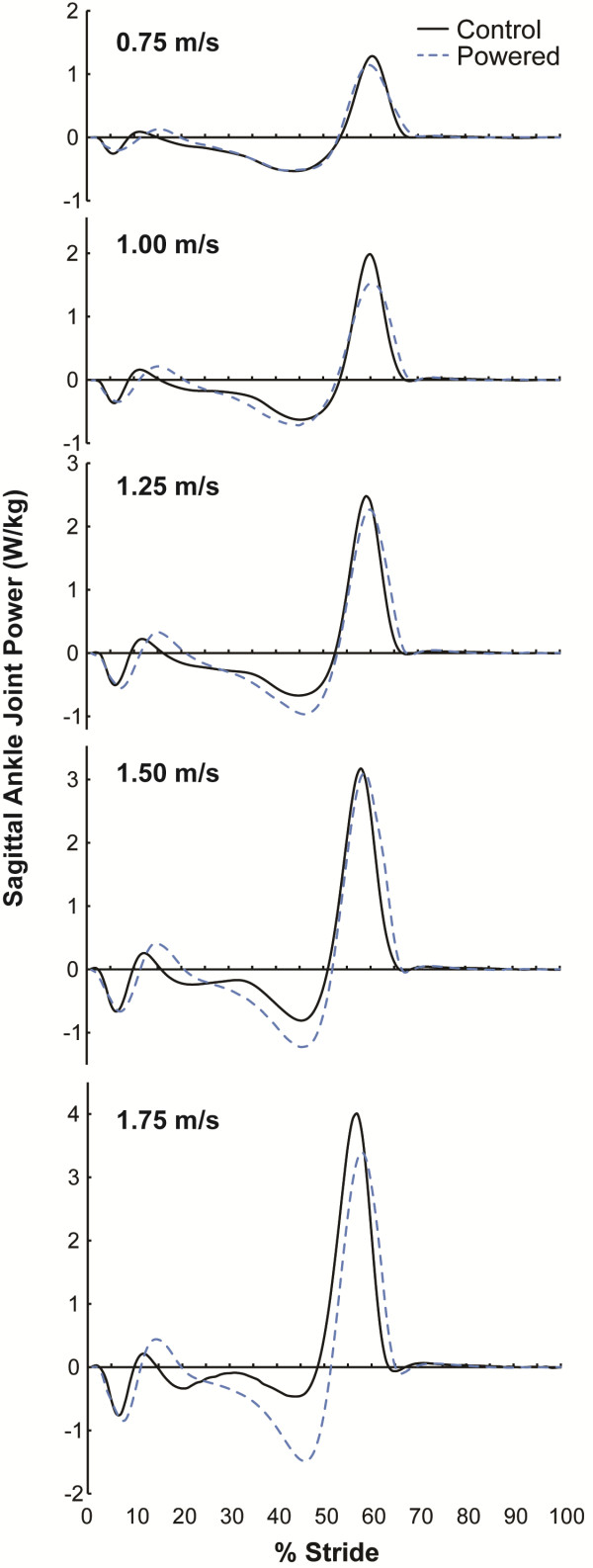
**Average powered prosthetic and biological sagittal ankle joint power.** Blue dashed lines represent prosthetic ankle joint power of the affected leg while subjects walked using the powered prosthesis (Powered). Black lines represent ankle joint power of non-amputees (Control). The average of three steps from all subjects is shown. Data are plotted versus percentage of a stride, where 0% occurs at heel strike.

Our results support the notion that greater prosthetic ankle work and power are associated with reductions in the first EAM peak on the unaffected knee. Similar to Morgenroth et al. [39], who examined the effects of different passive-elastic prostheses on the unaffected knee EAM, we also found that a prosthesis that performs more net positive work results in a lower unaffected leg first peak knee EAM (Figure 3). We calculated prosthetic ankle work during the entire stance phase, whereas Morgenroth et al. [39] calculated prosthetic ankle work only during the push-off phase of the gait cycle. In distinction to Morgenroth et al. [39], we compared knee EAM across a range of speeds and found statistical differences in peak EAM when subjects used the powered ankle-foot prosthesis compared to their own prescribed passive-elastic prosthesis at the two fastest walking speeds. Herr & Grabowski [38] found that subjects with an amputation using a powered ankle-foot prosthesis prefer to walk at 1.42 m/s, equivalent to the preferred speed of non-amputees, and 20% faster than their preferred speed when they used a passive-elastic prosthesis. Thus a significant reduction in peak EAM at a walking speed of 1.50 m/s has the potential to decrease the risk of knee osteoarthritis. Future research is warranted to systematically determine the effects of prosthetic ankle power and net positive ankle work on the unaffected knee EAM.

Previous research that measured the effect of lateral wedge insoles on a population with knee osteoarthritis has argued that a decrease of 5-7% in peak knee EAM may have significant clinical implications [51]. Though not statistically different, we found that the unaffected leg peak knee EAM of subjects using a powered prosthesis was 5.1 and 5.5% lower compared to using their own passive-elastic prosthesis at 0.75 and 1.25 m/s, respectively, and that the loading rate of knee EAM was more than 5% lower at all speeds when subjects used a powered compared to a passive-elastic prosthesis. Thus, use of a powered ankle-foot prosthesis could have important clinical implications by lowering unaffected leg knee moments and thereby reducing the risk of knee osteoarthritis.

Parameter tuning is a critical step in the setup procedure for the powered ankle-foot prosthesis. During tuning, we adjusted the amount of powered plantar flexion, the timing of powered plantar flexion, and the stiffness of the device during controlled plantar flexion until normative values of ankle toe-off angle, net prosthetic ankle work, and peak prosthetic ankle power were achieved (Table 2). We obtained data from the sensors on board the powered prosthesis and compared these values to normative biological data collected from previous studies [23,48]. Using these comparisons, we adjusted the control parameters to produce values within two standard deviations of the mean biological ankle data. The biological ankle data that we used for tuning net positive work [48] were not the same as the biological ankle data we obtained from our non-amputee subjects (Table 2), such that the values of net positive ankle work were all significantly greater for the powered prosthesis than for the biological ankle data we collected. We computed ankle toe-off angles and net positive work for the powered prosthesis using data obtained from the prosthetic ankle, and computed ankle toe-off angles and net positive work for non-amputees using inverse dynamics. This differential in the normative tuning data may have caused variability in our results. In the future, better tuning may yield more beneficial effects. Future studies are planned to understand the complexities inherent in tuning parameter optimization.

We found large percentage decreases in the loading rates of subjects using a powered compared to a passive-elastic prosthesis, but these differences were not significant. Therefore, our study may have been limited by a low number of participants (n=7) to detect differences in loading rates. In addition, the powered prosthesis accommodation period may have been too short. Our accommodation period is consistent with similar studies [39,52], however a longer accommodation period could allow people with an amputation to become more comfortable with the prosthesis, potentially decreasing their muscle co-contraction, adapting their mechanics and thus benefitting more from the powered prosthesis. A longer accommodation time could also allow the prosthesis to be re-tuned following any potential adaptation. Future studies are needed to determine the optimal accommodation times for and adaptations to novel prostheses.

We asked subjects to walk over ground at speeds within 0.10 m/s of five different target speeds. Though subjects walked within the speed range, they could have consistently walked faster or slower than the desired speed. We measured walking speeds from the position versus time of a marker placed over the 7^th^ cervical vertebrae and found that some walking speeds were significantly different between subject groups. Subjects with an amputation using a passive-elastic prosthesis walked at 0.76 (0.04), 1.03 (0.06), 1.25 (0.08), 1.53 (0.06), and 1.73 (0.05) m/s, subjects with an amputation using a powered prosthesis walked at 0.77 (0.05), 0.96 (0.06), 1.21 (0.04), 1.45 (0.04), and 1.67 (0.02) m/s, and non-amputees walked at 0.76 (0.04), 0.99 (0.05), 1.21 (0.05), 1.45 (0.06), and 1.70 (0.06) m/s. When using a passive-elastic prosthesis, subjects with an amputation walked significantly faster at the target speeds of 1.00, 1.25, and 1.75 m/s (P = 0.03, 0.004, and 0.04, respectively) compared to when they used a powered prosthesis. Additionally, subjects with an amputation using a passive-elastic prosthesis walked significantly faster at the target speed of 1.25 m/s compared to non-amputees (P = 0.04). These walking speed discrepancies were 6.8, 3.2, and 3.5% different on average for 1.00, 1.25, and 1.75 m/s, and are not likely to affect our results. However, future studies are planned that control walking speed and analyze the effects of using a powered prosthesis with an instrumented treadmill.

## Conclusions

A passive-elastic prosthesis cannot emulate normative biological function during the stance phase of walking; thus people with a lower-extremity amputation employ compensatory mechanics and have a higher incidence of musculoskeletal injury, specifically knee osteoarthritis in their unaffected leg. A biomimetic prosthesis could mitigate the risk of knee osteoarthritis by decreasing unaffected leg forces and knee moments. In this investigation, we found that when people with a unilateral transtibial amputation due to trauma and K3 level of ambulation used a powered ankle-foot prosthesis during level-ground walking over a range of speeds, they reduced the peak resultant force and knee adduction moment on their unaffected leg compared to when they used their own passive-elastic prosthesis. At the walking speed closest to preferred, subjects with an amputation using a powered ankle-foot prosthesis reduced their unaffected peak knee EAM by over 20%. A significant reduction in peak knee EAM has the potential to decrease the risk of knee osteoarthritis. Based on these results, we conclude that a biomimetic powered ankle-foot prosthesis could potentially limit musculoskeletal stress to the contralateral leg during walking, thus decreasing the risk of secondary injury in people with a lower-extremity amputation.

## Abbreviations

COM: Center of mass; EAM: External adduction moment; ANOVA: Analysis of variance; GRF: Ground reaction force.

## Competing interests

The authors declare that they have no competing interests.

## Authors’ contributions

AMG oversaw the experimental design, data acquisition, data processing, manuscript writing, and served as the PI. SD assisted with experiments and manuscript editing. All authors read and approved the final manuscript.
